# 

**Published:** 1889-10

**Authors:** 


### CONTENTS:

Article I.—A Case of Rhinolith and two Cases of a Tooth in the Nose. By Jonathan Wright, M. D. . 241

" II.—Neurectomy for Facial Neuralgia, with Recovery. By Dr. E. M. Magruder. . . . . . 247

" III —Some Notes on the Early Art of Extracting Teeth. By Frederick Sleep, L. D. S. . . . . 249

" IV.—The Preparation and Filling of Roots at one Sitting. By E. L. Clifford, D. D. S. . . . . 263

" V.—Dentition as Affected by Rickets. . . . . 276

### EDITORIAL.

Foreign Dentists in England. . . . . . . . . 278

Making every Child a Chemist. . . . . . . . . 278

### OBITUARY.

Dr. Philippe Ricord. . . . . . . . . . . . 281

### BIBLIOGRAPHICAL.

Artificial Crown and Bridge Work. . . . . . . 282

A Handbook of Dental Pathology for Students and Practitioners. 283

### MONTHLY SUMMARY.

A Real Elixir of Youth. . . . . . . . . . 284

Syphilitic Teeth. . . . . . . . . . . . 285

Nitrous Oxide Narcosis and the Blood. . . . . . . 286

Axioms in Prosthetic Dentistry. . . . . . . . . 287

Cocaine Hallucinations. . . . . . . . . . . 288

Death after taking Nitrous Oxide. . . . . . . . 288

